# Therapeutic Silencing of Centromere Protein X Ameliorates Hyperglycemia in Zebrafish and Mouse Models of Type 2 Diabetes Mellitus

**DOI:** 10.3389/fgene.2019.00693

**Published:** 2019-07-29

**Authors:** Liqing Zang, Yasuhito Shimada, Hiroko Nakayama, Wenbiao Chen, Ayaka Okamoto, Hiroyuki Koide, Naoto Oku, Takehisa Dewa, Masayuki Shiota, Norihiro Nishimura

**Affiliations:** ^1^Graduate School of Regional Innovation Studies, Mie University, Tsu, Japan; ^2^Mie University Zebrafish Drug Screening Center, Tsu, Japan; ^3^Department of Integrative Pharmacology, Mie University Graduate School of Medicine, Tsu, Japan; ^4^Department of Bioinformatics, University Advanced Science Research Promotion Centre, Tsu, Mie, Japan; ^5^Department of Molecular Physiology & Biophysics, Vanderbilt University School of Medicine, Nashville, TN, United States; ^6^Department of Medical Biochemistry, School of Pharmaceutical Sciences, University of Shizuoka, Shizuoka, Japan; ^7^Department of Frontier Materials, Graduate School of Engineering, Nagoya Institute of Technology, Nagoya, Japan; ^8^Department of Research Support Platform, Graduate School of Medicine, Osaka City University, Osaka, Japan

**Keywords:** centromere protein X, type 2 diabetes mellitus, insulin, zebrafish model, CRISPR/Cas9, gene silencing, therapeutic gene target

## Abstract

Type 2 diabetes mellitus (T2DM) is characterized by persistent hyperglycemia and is influenced by genetic and environmental factors. Optimum T2DM management involves early diagnosis and effective glucose-lowering therapies. Further research is warranted to improve our understanding of T2DM pathophysiology and reveal potential roles of genetic predisposition. We have previously developed an obesity-induced diabetic zebrafish model that shares common pathological pathways with humans and may be used to identify putative pharmacological targets of diabetes. Additionally, we have previously identified several candidate genes with altered expression in T2DM zebrafish. Here, we performed a small-scale zebrafish screening for these genes and discovered a new therapeutic target, centromere protein X (CENPX), which was further validated in a T2DM mouse model. In zebrafish, *cenpx* knockdown by morpholino or knockout by CRISPR/Cas9 system ameliorated overfeeding-induced hyperglycemia and upregulated insulin level. In T2DM mice, small-interfering RNA-mediated *Cenpx* knockdown decreased hyperglycemia and upregulated insulin synthesis in the pancreas. Gene expression analysis revealed insulin, mechanistic target of rapamycin, leptin, and insulin-like growth factor 1 pathway activation following *Cenpx* silencing in pancreas tissues. Thus, CENPX inhibition exerted antidiabetic effects *via* increased insulin expression and related pathways. Therefore, T2DM zebrafish may serve as a powerful tool in the discovery of new therapeutic gene targets.

## Introduction

Along with the epidemic of obesity, there is a parallel increase in the prevalence of diabetes worldwide (Haffner, 2006). Type 2 diabetes mellitus (T2DM) is characterized by persistent hyperglycemia and is primarily caused by pancreatic β-cell dysfunction and insulin resistance in target organs (Chatterjee et al., 2017). It is a complex disorder, and both genetic components, as well as environmental factors, are known to contribute to its pathogenesis (Ding et al., 2015). T2DM is regarded as an inevitably progressive condition with irreversible failure of beta cell function. Initiation of intensive glycemic control measures early in the diabetes course may be associated with a reduction of cardiovascular complications (Group et al., 2008). However, even tighter glucose control does not reduce the incidence of macrovascular diseases, such as nephropathy in patients with T2DM (Ukpds, 1998). Prevention of T2DM is a lifetime task and requires an integrated approach implemented right from the origin of the disease. Therefore, early detection and intervention of impaired glucose tolerance, the preliminary stage of T2DM, have attracted attention as a prophylactic treatment strategy.

T2DM is caused by the alterations of various functional genes owing to their own partial and additive effects, leading to a complex inheritance pattern (Tusie Luna, 2005). The identification of susceptible genes and genetic variants necessitates different methodological approaches. Over the past decade, hundreds of T2DM susceptible loci were captured using genome-wide association studies (GWAS) (Morris et al., 2012; Flannick and Florez, 2016). However, some researchers questioned the biological relevance of these variants in disease or their clinical utility for prognosis or treatment (Goldstein, 2009; Mcclellan and King, 2010). Transcriptomics, or the study of the complete set of RNA transcripts produced by the genome (e.g., *via* RNA sequencing), is a powerful tool to explore the association between genes and T2DM and through the correlation between the genotype and phenotype (Jenkinson et al., 2016). The strategies exploiting transcriptomics and animal models are expected to discover the novel genetic factors of diabetes that may be crucial for the identification of risk factors, diagnostic indicators (biomarkers), and new therapeutic targets.

Zebrafish is a well-established and widely used model system (Dooley and Zon, 2000) that offers several advantages, including characteristics of vertebrates, high fecundity, rapid development, transparent body in the embryo and larval stage, and a high degree of genetic, anatomical, and physiological similarities to humans. Zebrafish has been traditionally used in developmental biology (Kishi, 2011). In the last decade, it has become a valuable tool to model human diseases, including metabolic diseases (Santoriello and Zon, 2012; Zang et al., 2018). Three approaches are used for the development of a zebrafish obesity model, including an induced model, transgenic line, and mutant line. The induced model is the most common approach, which is carried out by treating zebrafish with a high-fat diet or over-nutrition (Zang et al., 2018). Researchers have designed high-fat zebrafish diets containing chicken egg yolk, vegetable oil, or lard and successfully generated obese zebrafishes (Meguro et al., 2015; Landgraf et al., 2017). Overfeeding with *Artemia* may also serve as a convenient method to induce obesity in zebrafish (Oka et al., 2010; Tainaka et al., 2011; Hiramitsu et al., 2014; Zang et al., 2014; Zang et al., 2015b). Additionally, zebrafish models of T2DM are also developed with glucose immersion, over-nutrition, and genetic modification (Zang et al., 2018). We have recently created a zebrafish model for both obesity and T2DM by over-nutrition and demonstrated its application in studying human diabetes (Zang et al., 2017). We further performed RNA deep sequencing (RNA-Seq) analysis and identified several candidate genes with altered expression patterns in T2DM zebrafish; these observations have not been reported in studies with humans and rodents (Zang et al., 2017). In the present study, we first carried out a small-scale knockdown screening of these candidate genes using morpholino (MO) antisense oligos to identify their relationships with T2DM. We next performed knockout and knockdown studies in zebrafish and mouse models of T2DM and elucidated that centromeric protein X (CENPX) may be a potential therapeutic target in diabetes.

## Materials and Methods

### Zebrafish Strains and Maintenance

AB strain and *Tg(−1.0ins:EGFP)* *^sc1^* strain (referred to as *ins-EGFP*) (Diiorio et al., 2002) (the Zebrafish International Research Centre, Eugene, OR, USA), and a skeletal muscle insulin-resistant zebrafish transgenic line (zMIR; *Tg(acta1:dnIGF1R-EGFP)*) (Maddison et al., 2015) were maintained at 28°C with a light/dark cycle of 14/10 h (Westerfield, 2007).

### Over-Nutrition-Induced T2DM Zebrafish Model

The T2DM zebrafish model was induced as previously described (Zang et al., 2017). In brief, male healthy adult zebrafishes (4–6 months old) were assigned to either an overfeeding (diet-induced obesity; DIO) or a control group (non-DIO). DIO zebrafishes were fed 120 mg/fish of Otohime B2 (Marubeni Nisshin Feed, Tokyo, Japan) each day (divided into six feedings), whereas non-DIO zebrafishes were fed 20 mg/fish once a day every day. The body weight was measured weekly during the overfeeding treatment. At the experimental endpoint, the blood sample was collected and the fasting blood glucose (FBG) level was measured as previously described (Zang et al., 2013; Zang et al., 2015a).

### Morpholino Treatment

The sequences of antisense vivo-morpholino (MO; Gene Tools, Philomath, OR, USA) for *cenpx* and *hmox1* are provided in **Supplementary Table S1**. MO was intraperitoneally injected at a dose of 15 mg/kg fish weight twice a week during the 8-week experiment period. Additional methods details are given in the Supplementary Material. For the quantitative analysis of insulin level, *ins-EGFP* zebrafishes were treated with *cenpx* vivo-MO for 4 weeks. EGFP signaling was quantified using ImageJ software (National Institutes of Health, Bethesda, MD, USA) as previously described (Zang et al., 2017).

### *cenpx* sgRNA Microinjection

Zebrafish *cenpx* mutants were generated using a vector-free method of Clustered Regularly Interspaced Short Palindromic Repeats (CRISPR)-Cas9 mutagenesis, with some modifications in a previously described report (Varshney et al., 2015). Additional methods details are given in the Supplementary Material. For sgRNA microinjection, zMIR strain embryo was injected with approximately 1 nl solution containing 40–50 pg of *cenpx* sgRNA, 250 pg of Cas9 protein (PNA Bio, CA, USA), and phenol red at single-cell stage. Injected embryos were raised to adulthood and the adult zebrafishes were assigned to either an overfeeding or a control group. The overfeeding experiment was performed as described above.

### Mouse Experiments

Ten-week-old male NSY/Hos mice, an inbred animal model with spontaneous development of T2DM, were purchased from SLC (Shizuoka, Japan). The mice were housed in groups of five in one cage and fed with basal diet (CE-7; CLEA Japan, Tokyo, Japan) or high-fat diet (HFD; 58Y1, Test Diet, Richmond, IN, USA) to induce obesity and diabetes. During the feeding experiment, body weight was measured once a week. Mice were kept fasting for 14 h before withdrawing blood samples for the measurement of fasting levels of blood glucose.

### Preparation of Small-Interfering RNA (siRNA)-Lipoplexes and *In Vivo* Gene Silencing

The siRNA against mouse *Cenpx* and a scrambled siRNA (negative control) were purchased from Hokkaido System Science Co. (Hokkaido, Japan). The sequences of siRNAs are shown in **Supplementary Table S1**. The siRNAs were entrapped between the lipid layers of multilayered liposomes by freeze-thawing of lipoplexes comprising polycation liposomes (PCLs) and siRNAs, as previously described (Koide et al., 2016). Freeze-dried siRNA-lipoplexes were diluted with distilled water, and 200 µl of siRNA-lipoplexes (containing 50 µg of siRNAs) or vehicle (0.3 M sucrose) was injected *via* the tail vein to each NSY/Hos mouse. The siRNA was administered twice per week for 4 weeks. At the end of the experiment, the mice were euthanized with CO_2_ gas, and liver and pancreas tissues were harvested for immunohistochemistry (IHC) and quantitative polymerase chain reaction (qPCR) analyses.

### Fluorescent Immunohistochemistry Staining

Mouse pancreas tissues were fixed with 4% paraformaldehyde and embedded in paraffin. Sections (4 µm) were rehydrated and subjected to antigen retrieval by heating in 0.5% Immunosaver (Nisshin EM, Tokyo, Japan) at 98°C for 45 min. The sections were incubated with a primary antibody against insulin (1:200; Abcam, ab7842, Cambridge, UK) overnight at 4°C. After rinsing with Tris-buffered saline (TBS), the sections were incubated with a fluorescence-conjugated secondary antibody (Goat Anti-Guinea pig IgG H&L, Alexa Fluor^®^ 488; Abcam, ab15015) at room temperature (20°C) for 1 h and mounted with Prolong Glass Antifade Mountant (Abcam, P36984). Images were captured using a BZ-X710 fluorescence microscope (GFP filter; Keyence, Tokyo, Japan). The fluorescence signal of insulin was quantified and normalized with the related islets of Langerhans area using ImageJ.

### Insulin Signaling Pathway Analysis by a qPCR Array

Total RNA purification and cDNA synthesis from mouse pancreas tissues were performed as previously described (Zang et al., 2017). A PCR screening array specific for mouse insulin signaling was used (RT2 Profiler PCR Array; QIAGEN PAMM-030ZC) to examine the expression of 89 genes (including five housekeeping genes; **Supplementary Table S2**). qPCR analysis was performed with ABI StepOnePlus Real-Time PCR System using SYBR Green. Relative gene expression was determined using the ΔC_T_ calculation method. After statistical tests, we performed sub-network enrichment analysis using Pathway Studio 9.0 (Elsevier, Amsterdam, Netherlands) according to our previous study (Zang et al., 2017).

### Statistical Analysis

All results are presented as means with their standard errors (SEs). Data were analyzed with GraphPad Prism. *P-value* ≤ 0.05 was considered statistically significant. Statistical analyses were carried out by Student’s *t*-test or one-way analysis of variance (ANOVA) with the Bonferroni–Dunn multiple comparison procedure, depending on the number of comparisons.

## Results

### MO-Mediated Knockdown Screening Identifies *cenpx* as a Novel Therapeutic Candidate in Diabetic Zebrafish

Based on the altered genes expression in hepatopancreas tissues of T2DM zebrafish (Zang et al., 2017), we performed qPCR analysis on the selective target genes for the subsequent experiments (**Supplementary Table S3**). We next performed knockdown screening to AB strain zebrafish using vivo-MO to investigate candidates that could reverse blood glucose elevation in T2DM fish. As a result, the knockdown of the genes encoding centromeric protein X (*cenpx*) and heme oxygenase 1 (*hmox1*) significantly (*P* < 0.05) reduced the overfeeding-induced elevation in blood glucose level (**Supplementary Figure S1**). We focused on *cenpx* and validated its potential as a novel therapeutic candidate gene for diabetes and *homx1* was used as a positive control (Jais et al., 2014).

**Figure 1:**
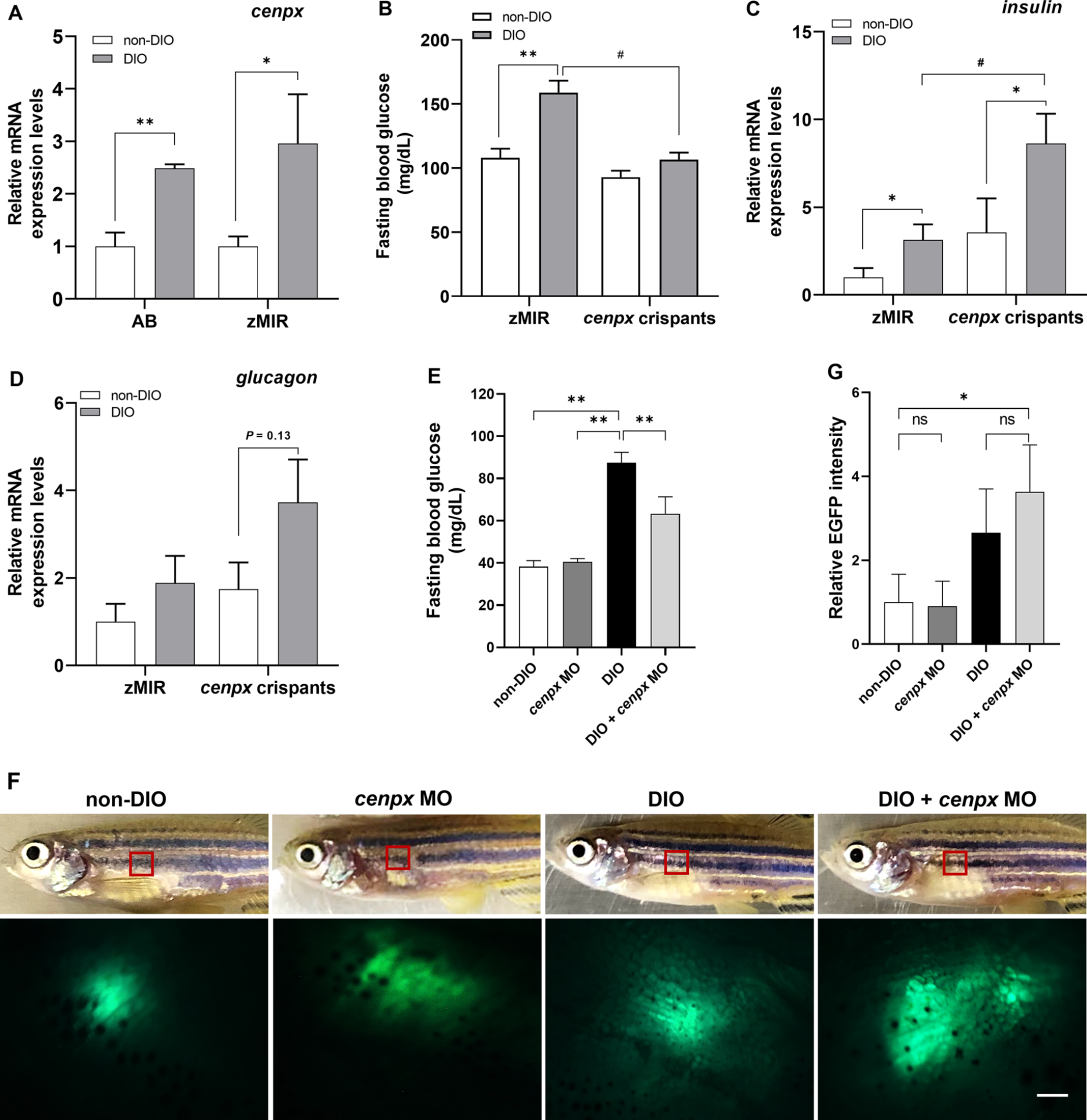
Deletion of *cenpx* gene suppressed hyperglycemia in diabetic zebrafish. **(A)** The relative mRNA level of *cenpx* in the hepatopancreas tissue of AB and zMIR zebrafish. Data are shown as relative levels of *cenpx* mRNA of AB and zMIR strains to that in their corresponding non-DIO controls. n = 5. ^*^ *P* < 0.05, ^**^ *P* < 0.01 versus the non-DIO group. **(B)** The fasting blood glucose levels in zMIR zebrafish and *cenpx* crispants after overfeeding treatment. n = 5. ^**^ *P* < 0.01 versus the non-DIO group; ^#^ *P* < 0.05 versus the DIO group. **(C, D)** The mRNA levels of *insulin* and *glucagon* in *cenpx* crispants. RNA was isolated from the hepatopancreas of zebrafishes from overfeeding and normal feeding groups. The mRNA levels of *insulin* and *glucagon* were compared with that of amylase as a marker for the endocrine pancreas. Data are shown as the relative expression level of *insulin* and *glucagon* mRNA to that in non-DIO zMIR. n = 5. ^*^ *P* < 0.05 *versus* the non-DIO group; ^#^ *P* < 0.05 *versus* the DIO zMIR group. **(E)** Fasting blood glucose levels in MO-treated *ins*-*EGFP* zebrafish after 4 weeks of normal feeding or overfeeding. Fishes were treated with *cenpx* MO twice a week. n = 5. **P < 0.01 *versus* the DIO group; **(F)** Upper panel are the bright field of *ins*-*EGFP* zebrafishes with or without i.p. injection of *cenpx* MO in DIO. Lower panel are EGFP levels in the exocrine pancreas areas that were monitored by fluorescence stereoscopic microscopy. The red box marks the region displayed in the fluorescence image. Scale bar = 0.5 mm. **(G)** Graph of relative EGFP intensities from lower panel of F. n = 5. ^*^ *P* < 0.05 *versus* the non-DIO group.

The T2DM model zebrafish we developed previously was used as wild-type AB strain (Zang et al., 2017). The highest FBG level was approximately 95 ± 9 mg/dl. However, zMIR, a transgenic line of insulin resistance (Maddison et al., 2015), shows higher FBG levels (171 ± 39 mg/dl) after overfeeding to induce T2DM compared with that of the AB strain (*P* < 0.05). For this reason, we selected zMIR as a strong T2DM zebrafish model in the following experiments. We next confirmed the expression of *cenpx* in wild-type (AB) and zMIR T2DM fishes after overfeeding. As shown in **Figure 1A**, the relative mRNA expression level of *cenpx* in AB and zMIR overfed zebrafish (DIO) was 2.5- and 3-fold higher as compared with their corresponding normally fed zebrafishes (non-DIO), respectively. The basal expression levels of *cenpx* in the non-DIO zMIR group had no statistically significant difference when compared with non-DIO AB group.

### Knockout of *cenpx* Ameliorates Diabetes in Zebrafish and Upregulates Insulin Level

To investigate the relationship between *cenpx* and hyperglycemia, we designed and synthesized three single guide RNAs (sgRNAs) targeted to *cenpx* to generate gene disruption in F0 zebrafish crispants. After microinjection of the sgRNAs combined with Cas9 protein into zMIR strain, the genome DNA from 24 hpf (hour post fertilization) embryos was extracted and examined for genome editing activity using a heteroduplex mobility assay (Ota et al., 2013). The most effective sgRNA that triggered mutations was sgRNA1 (**Supplementary Figure S2**). We then selected *cenpx* sgRNA1 to produce F0 founders. These fishes were raised till the adult stage and then overfed for 4 weeks along with un-injected zMIR zebrafish. The un-injected zMIR zebrafish showed a significant increase in the FBG level (**Figure 1B**). On the contrary, *cenpx* crispants showed no elevation in FBG level as compared with the fish on a normal diet. Real-time qPCR analysis using the RNA isolated from the hepatopancreas of zMIR revealed a significant increase in the mRNA level of the *insulin* gene in the overfed zMIR zebrafish as compared with the fish fed with a normal diet (*P* < 0.05, **Figure 1C**). Analysis of the *insulin* mRNA levels in *cenpx* crispants showed a statistically significant increase of 2.4-fold in DIO *cenpx* crispants when compared with that in the non-DIO *cenpx* crispants (*P* < 0.05). We also found a significantly higher *insulin* expression level in DIO *cenpx* crispants than in the DIO zMIR group (*P* < 0.05). Additionally, the glucagon expression showed no statistically significant difference in DIO *cenpx* crispants compared with non-DIO *cenpx* crispants (*P* = 0.13; **Figure 1D**).

To confirm the overproduction of insulin following silencing of the *cenpx*, we performed intraperitoneal injection of *cenpx* vivo-MO in the *ins*-*EGFP* zebrafish twice a week for 4 weeks and monitored EGFP expression in the pancreas using fluorescence stereoscopic microscopy. The increase in blood glucose levels induced by overfeeding was suppressed upon *cenpx* MO treatment (*P* < 0.01), consistent with the observation in AB strain (**Figure 1E**). The EGFP-positive areas in the pancreas increased in *cenpx* MO-injected DIO group as compared with the non-DIO group (*P* < 0.05; **Figures 1F, G**). There was no significant difference between non-DIO and *cenpx* MO groups on a non-DIO background, or between DIO and DIO + *cenpx* MO group.

### Knockdown of Cenpx Ameliorates Hyperglycemia in Mouse T2DM Model

**Figure 2:**
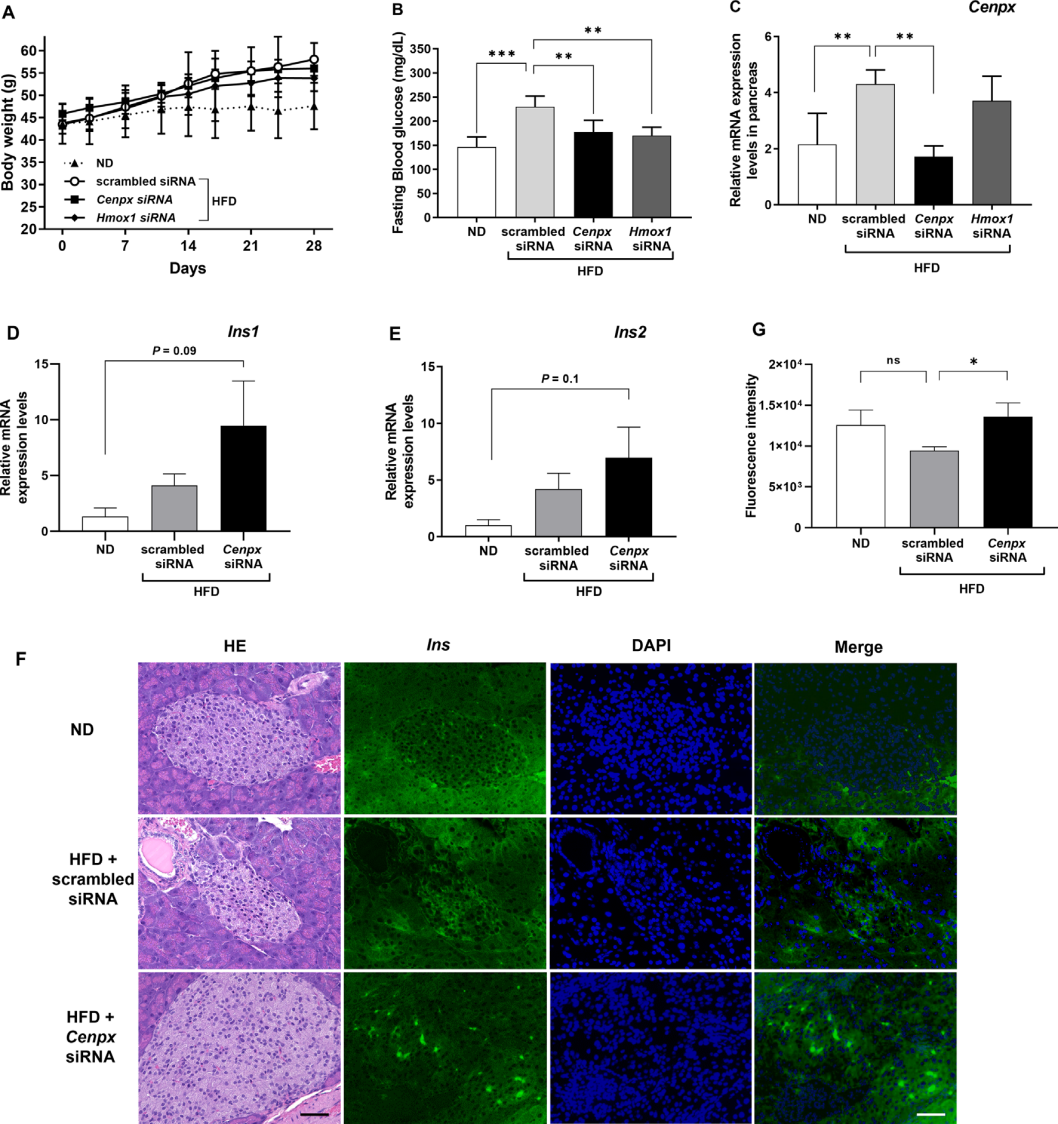
Silencing of *Cenpx* ameliorates elevated blood glucose levels in a diabetes mouse model. **(A)** Changes in body weight. **(B)** Changes in fasting blood glucose levels. **(C)** Relative mRNA levels in the pancreas from the HFD mice injected with scrambled-, *Cenpx-, and Hmox1*-siRNAs. The mRNA levels of **(D)** Insulin 1 (*Ins1*) and **(E)** Insulin 2 (*Ins2*) in HFD mouse treated with *Cenpx*-siRNA (n = 5). **(F)** H&E and fluorescent IHC staining for Insulin in ND and HFD mice injected with scrambled siRNA and *Cenpx* siRNA in the pancreas tissue. Scale bar = 50 µm. **(G)** Quantification of the fluorescence intensity of insulin in the islets of Langerhans, which was normalized by the related area. n = 10; ns, not significant; *P < 0.05, **P < 0.01, ***P < 0.001 *versus* the scrambled siRNA group.

We conducted knockdown of *Cenpx* using siRNA technique in NSY/Hos mice, a spontaneous model of T2DM (Ueda et al., 1995). We designed and synthesized three siRNAs directed against mouse *Cenpx* gene and investigated the knockdown efficiency *in vitro* using Hepa 1-6 cells (**Supplementary Figure S3**). Real-time qPCR analysis showed the siRNA2 effectively inhibited the expression of *Cenpx*, and therefore siRNA2 was selected for further *in vivo* experiments. For positive control, *Hmox1* siRNA was synthesized according to a previous report (Zhang et al., 2004). siRNA with a scrambled sequence based on *Cenpx* siRNA was designed and used as the negative control. For siRNA knockdown experiment, mice were fed a normal diet (ND) and a high fat-diet (HFD) and administered with scrambled-, *Cenpx-*, and *Hmox1-*siRNAs for 4 weeks. No significant differences in FBG levels and *Cenpx* mRNA levels in pancreas tissue were found between ND and ND with scrambled siRNA treatment groups, and between HFD and HFD with scrambled siRNA treatment groups (**Supplementary Figure S4**). The three siRNA-administrated groups fed with HFD exhibited a significant (*P* < 0.05) increase in body weight as compared with the ND group. No significant difference in body weight was observed between scrambled-, *Cenpx-*, and *Hmox1-*siRNA treatment groups (**Figure 2A**). The FBG levels significantly increased in scrambled siRNA-injected mice fed with HFD (230 ± 10 mg/dl) as compared with the mice in the ND group (146 ± 9 mg/dl) (**Figure 2B**). In contrast, the FBG levels of *Cenpx* siRNA- and *Hmox1* siRNA-injected mice fed with HFD were 178 ± 11 and 170 ± 8 mg/dl, respectively, which showed a significant decrease compared to that of scrambled siRNA-injected mice fed with HFD (*P* < 0.05). Quantitative PCR analysis revealed that *Cenpx* siRNA significantly inhibited the *Cenpx* mRNA expression level in the pancreas (**Figure 2C**); however, no silencing effect was observed in the liver tissue (**Supplementary Figure S5**).

Consistent with the results observed in the zebrafish model (**Figure 1C**), the expression of the gene encoding insulin 1 (*Ins1*) had a trend towards upregulation and that of insulin 2 (*Ins2*) had no change after *Cenpx* siRNA administration in the mouse pancreas compared with that of the ND group (*P* = 0.09 and *P* = 0.1, respectively; **Figure 2D, E**). There was no statistically significant difference between scrambled siRNA treated HFD mice and *Cenpx* siRNA treated HFD mice. The results of fluorescent IHC staining using insulin antibody are shown in **Figure 2F**. We quantified the fluorescent signal of insulin in each islet of Langerhans and normalized with the related area (**Figure 2G**). No significant difference was found in insulin level between ND and scrambled siRNA group with HFD treatment. However, we observed a significant increase in the expression of insulin protein in the pancreas tissue of mice from *Cenpx* siRNA administration HFD group as compared with the mice from the scrambled siRNA HFD group (*P* < 0.05).

### Gene Expression Analysis in the Pancreas of *Cenpx* Knockdown T2DM Mice

**Figure 3:**
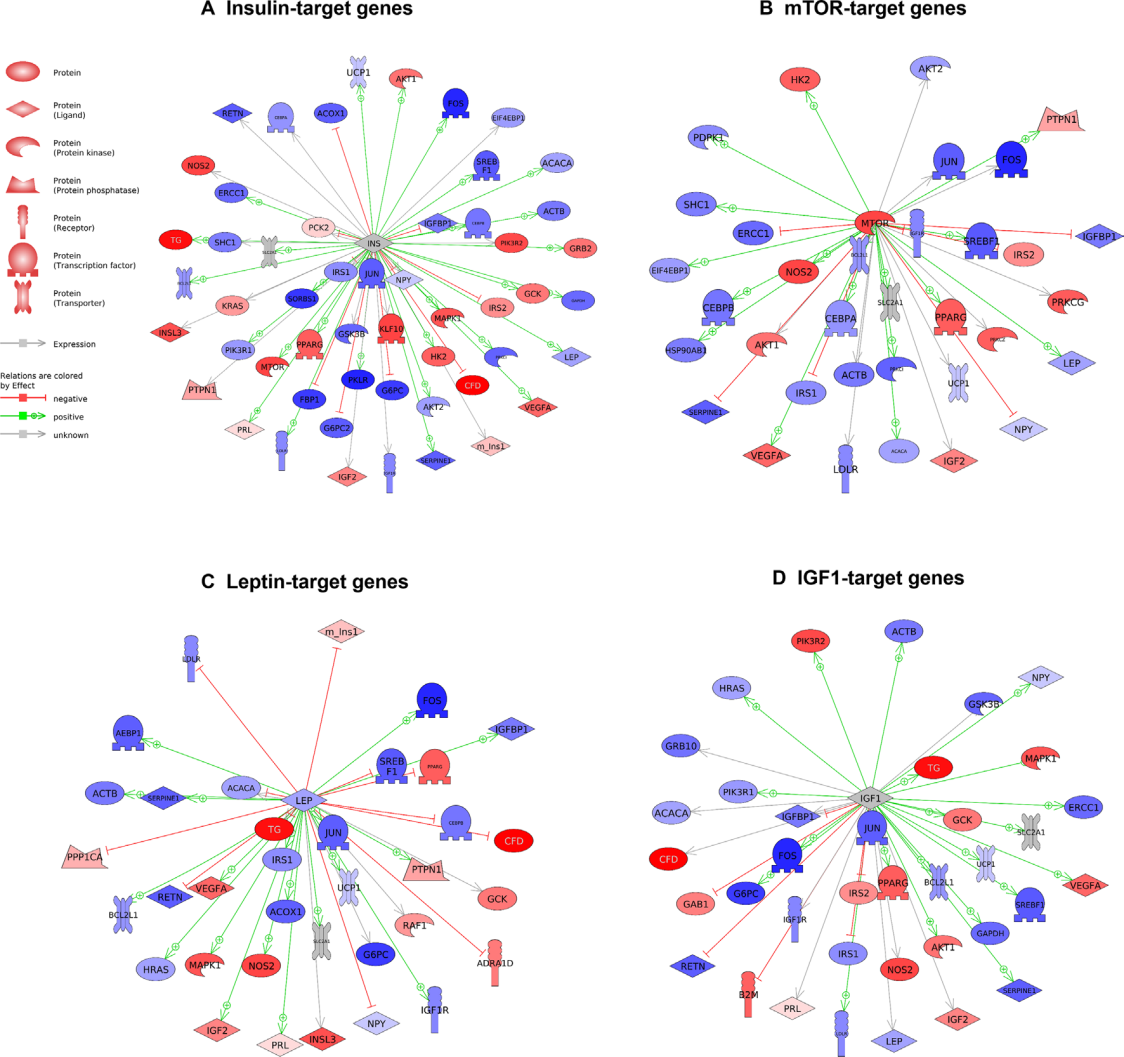
Pathways activated by *Cenpx* knockdown in the mouse pancreas tissue. **(A)** Insulin-target gene pathway. **(B)** mTOR-target gene pathway. **(C)** Leptin-target gene pathway. **(D)** IGF1-target gene pathway. Red and blue denote genes with increased and decreased expression, respectively, in *Cenpx* knockdown in HFD mice as compared with control HFD mice. Grey indicates the genes that were undetected in the gene expression analysis.

Gene expression analysis using a qPCR array for the insulin signaling pathway was performed to investigate the mechanism in the pancreas tissues of *Cenpx* siRNA-injected NSY/Hos mice fed with a high-fat diet. Of 84 genes, 17 and 20 genes were upregulated (>1.5-fold) and downregulated (<0.75-fold), respectively, following knockdown of *Cenpx* by siRNA in T2DM mice as compared with the HFD treated control mice (T2DM mice) (**Table 1**). We performed a sub-network enrichment analysis for differentially expressed target genes (Kotelnikova et al., 2010) and found several pathways that were altered after *Cenpx* knockdown (**Supplementary Table S4**). The top four pathways, including insulin, mammalian target of rapamycin complex 1 (mTOR), leptin, and insulin-like growth factor 1 (IGF1) are shown in **Figure 3**. Of these, INSULIN signaling pathway was upregulated by knockdown of *Cenpx* in T2DM mice as compared with the T2DM mice (**Supplementary Figure S6A**).

**Table 1:** Fold change in the levels of gene expression in pancreas of *Cenpx* siRNA-treated T2DM mouse compared to those in pancreas of a T2DM control mouse.

Gene symbol	Description	Refseq	*Cenpx* siRNA vs. T2DM
*Cfd*	Complement factor D (adipsin)	NM_013459	5.74
*Tg*	Thyroglobulin	NM_009375	4.42
*Dok3*	Docking protein 3	NM_013739	4.24
*Dusp14*	Dual specificity phosphatase 14	NM_019819	2.95
*Nck1*	Non-catalytic region of tyrosine kinase adaptor protein 1	NM_010878	2.13
*Klf10*	Kruppel-like factor 10	NM_013692	2.07
*Mtor*	Mechanistic target of rapamycin (serine/threonine kinase)	NM_020009	2.05
*Nos2*	Nitric oxide synthase 2, inducible	NM_001313921	2.01
*Pik3r2*	Phosphatidylinositol 3-kinase, regulatory subunit, polypeptide 2 (p85 beta)	NM_008841	1.95
*Insl3*	Insulin-like 3	NM_013564	1.82
*Mapk1*	Mitogen-activated protein kinase 1	NM_011949	1.75
*Prkcg*	Protein kinase C, gamma	NM_011102	1.67
*Pparg*	Peroxisome proliferator activated receptor gamma	NM_011146	1.64
*Prkcz*	Protein kinase C, zeta	NM_008860	1.63
*Braf*	Braf transforming gene	NM_139294	1.62
*Hk2*	Hexokinase 2	NM_013820	1.62
*Vegfa*	Vascular endothelial growth factor A	NM_009505	1.54
*Ercc1*	Excision repair cross-complementing rodent repair deficiency, complementation group 1	NM_007948	0.74
*Gsk3b*	Glycogen synthase kinase 3 beta	NM_019827	0.71
*Prkci*	Protein kinase C, iota	NM_008857	0.69
*Acox1*	Acyl-Coenzyme A oxidase 1, palmitoyl	NM_015729	0.67
*Aebp1*	AE binding protein 1	NM_009636	0.66
*Serpine1*	Serine (or cysteine) peptidase inhibitor, clade E, member 1	NM_008871	0.66
*Jun*	Jun oncogene	NM_010591	0.65
*G6pc2*	Glucose-6-phosphatase, catalytic, 2	NM_021331	0.65
*Retn*	Resistin	NM_022984	0.65
*Srebf1*	Sterol regulatory element binding transcription factor 1	NM_011480	0.63
*Akt3*	Thymoma viral proto-oncogene 3	NM_011785	0.62
*Gpd1*	Glycerol-3-phosphate dehydrogenase 1 (soluble)	NM_010271	0.61
*Slc2a1*	Solute carrier family 2 (facilitated glucose transporter), member 1	NM_011400	0.60
*Map2k1*	Mitogen-activated protein kinase kinase 1	NM_008927	0.55
*G6pc*	Glucose-6-phosphatase, catalytic	NM_008061	0.40
*Fbp1*	Fructose bisphosphatase 1	NM_019395	0.40
*Slc27a4*	Solute carrier family 27 (fatty acid transporter), member 4	NM_011989	0.40
*Sorbs1*	Sorbin and SH3 domain containing 1	NM_009166	0.37
*Fos*	FBJ osteosarcoma oncogene	NM_010234	0.26
*Pklr*	Pyruvate kinase liver and red blood cell	NM_013631	0.24

## Discussion

Knockdown of *Cenpx* by siRNA in mice significantly decreased the levels of the *Cenpx* mRNA in the pancreas, but there was no change in the liver tissue (**Figure 2C** and **Supplementary Figure S5**). These data suggest the specificity of siRNA for pancreatic tissue, most likely due to pharmacokinetic properties of siRNA. Huang and colleagues have reported that intravenously administered siRNA exhibits remarkable accumulation in the glandular tissues, including the pancreas (Huang et al., 2016). The half-life of siRNA in the pancreas was 2.3-fold longer than that in liver tissue (30.3 h vs. 13 h, respectively). Therefore, to sustain the effect of siRNA-mediated knockdown, we administered the siRNA twice per week, and the effects of *Cenpx* siRNA in the liver were hardly observed due to a shorter half-life of siRNA in liver than pancreas tissues.

Current treatment modalities for T2DM mainly focus on lowering blood glucose level *via* lifestyle modification or anti-diabetic drugs that stimulate insulin secretion from the pancreas or enhance the peripheral tissue sensitivity (Chatterjee et al., 2017). However, patients with diabetes may require multiple medications to avoid chronic hyperglycemia-caused complications for considerable heterogeneity in the therapeutic response (Maccallum, 2014). Therefore, clarification of the onset mechanism of T2DM and identification of novel therapeutic genes are desirable for more effective and safer treatment of this disease. GWAS is a primary approach for the identification of genetic variants associated with disease risk (Mccarthy et al., 2008). In the past decade, 144 genetic variants at 129 susceptibility loci have been reported to be associated with T2DM (Morris et al., 2012; Flannick and Florez, 2016). However, only a small proportion (∼10%) from this large number of T2DM-associated variants identified by GWAS could explain the heritability of T2DM, suggesting that much of the variants are still undiscovered. Recently, with the high cost and low clinical utility of GWAS (Goldstein, 2009; Mcclellan and King, 2010), new strategies that could identify the causal variants of T2DM were proposed, including next-generation sequencing (NGS) studies and/or post-GWAS experiments, including gene function study, animal models, and clinical studies (Mcclellan and King, 2010). Here, we combined transcriptomic study (NGS), gene function study (gene expression analysis, CRISPR/Cas9, and RNA interference technique) and animal models to discover new predictors of T2DM development. As a result, CENPX was identified as a novel therapeutic target for T2DM. Our results demonstrate the suitability of this combinational genetic study for new drug target discovery in T2DM and resolution of the questions disputed with GWAS.

CENPX, also referred to as FANCM-interacting histone-fold protein 2 (MHF2), is localized at the centromeres throughout the cell cycle (Amano et al., 2009). It was recently classified as an integral component of the Fanconi anemia (FA) core complex, which is required for DNA damage response and repair (Yan et al., 2010). CENPX is a component of three different complexes, namely: 1) CENPS-CENPX complex, essential for the stable assembly of the outer kinetochore (Amano et al., 2009; Perpelescu and Fukagawa, 2011); 2) CENP-T-W-S-X complex, that binds to supercoiled DNA and plays an important role in kinetochore assembly (Nishino et al., 2012); 3) CENPX and CENPS, Fanconi Anemia M (FANCM)-associated proteins (Singh et al., 2010; Yan et al., 2010; Nishino et al., 2012). MHF1 (CENPS) and MHF2 form MHF1–MHF2 heterodimers and interact with each other to form an (MHF1–MHF2)_2_ tetramer that preferentially binds to branched DNA and interacts with FANCM to facilitate the repair of branched DNA (Fox et al., 2014; Zafar et al., 2017). Taken together, CENPX is essential to protect genome stability and plays a role in DNA damage repair. Although DNA damage is thought to be a major contributing factor for T2DM, little is known about the potential contribution of DNA repair disturbance in diabetes (Blasiak et al., 2004; Shimizu et al., 2014). In T2DM, evidence of increased oxidative DNA damage and decreased DNA repair activity has been reported (Tabak et al., 2011; Manoel-Caetano et al., 2012). However, our results revealed an increase in *cenpx* expression level in diabetic zebrafish, and the knockdown or knockout of *Cenpx* expression exerts a protective effect in diabetic zebrafish and mouse models. We hypothesize that CENPX may participate in T2DM by altering the expression of the genes responsible for glucose homeostasis rather than performing any role in DNA repair. For instance, insulin action may be negatively regulated by CENPX under a hyperglycemic state. Therefore, CENPX deletion (or reduced expression) may result in the upregulation of insulin expression and consequently activation of downstream pathways.

To clarify our hypothesis, we predicted the therapeutic pathways induced by *Cenpx* knockdown in the pancreas tissue of T2DM mice using qPCR array. We found insulin signaling to be a major pathway (**Figure 1**) as per the results observed in zebrafish, though other obesity- or diabetes-related pathways may also be affected following *Cenpx* knockdown. The mTOR pathway, the second most affected pathway (**Supplementary Table S4**), is known to form two distinct multiprotein complexes known as mTOR complex 1 (mTORC1) and mTOR complex 2 (mTORC2) (Sengupta et al., 2010; Oh and Jacinto, 2011). It is shown to be highly involved in insulin signaling (Yoon, 2017). The mTOR signaling pathway is dysregulated in human T2DM (Um et al., 2004; Newgard et al., 2009), and the loss of mTORC1 signaling results in the impairment of β-cell homeostasis and insulin processing (Blandino-Rosano et al., 2017). Intriguingly, we observed that the EIF4EBP2 pathway acting downstream of mTORC1, which is also a known regulator of beta cell proliferation in the pancreas, was also upregulated after *Cenpx* knockdown in T2DM mice (**Supplementary Figure S6B**) (Blandino-Rosano et al., 2017). Consistent with these results in mice, *cenpx* knockdown (morphant) increased the number of beta cells in zebrafish (**Figure 1**). Leptin and IGF1 pathways are strongly connected with insulin signaling (Hakuno and Takahashi, 2018; Marques-Oliveira et al., 2018) and were upregulated after *Cenpx* knockdown in T2DM mice. These transcriptome results are reasonably associated with the phenotypes of CENPX knockdown mice, indicating that the pathophysiological contribution of CENPX would be common in all vertebrate animals. However, the direct relationship between CENPX expression and these pathways is still unclear. Future studies are warranted to confirm these findings to provide a better understanding of the research in this field.

In the present study, we discovered CENPX as a new therapeutic target against T2DM using zebrafish and mouse models. We performed *cenpx* knockout experiments using CRISPR/Cas9 genome editing technology in T2DM zebrafish and *Cenpx* knockdown experiments using siRNA technique in the T2DM mouse model. Inhibition of CENPX expression in zebrafish and mouse diabetic models led to the amelioration of hyperglycemia through the induction of insulin secretion. To the best of our knowledge, no report has been published on the assessment of expression or activity of CENPX in obesity, glucose homeostasis, and diabetes mellitus. Our findings highlight CENPX inhibition as a potential therapeutic strategy for diabetes control.

## Data Availability

The raw data supporting the conclusions of this manuscript will be made available by the authors, without undue reservation, to any qualified researcher.

## Ethics Statement

All animal procedures were approved by the Ethics Committee of Mie University and were performed according to the Japanese Animal Welfare Regulation “Act on Welfare and Management of Animals” (Ministry of Environment of Japan) and complied with international guidelines.

## Author Contributions

LZ and YS conceived and designed the research; performed the experiments; analyzed the data; interpreted the results of the experiments; prepared the figures; and drafted, edited, revised, and approved the final version of the manuscript. HN, AO, HK, NO, DT, and MS performed the experiments and analyzed the data. WC revised and approved the final version of the manuscript. NN takes sole responsibility for the integrity, accuracy, and analysis of the data, and has full access to the data.

## Funding

This work was supported by JSPS KAKENHI (grant numbers 15K19074, 15KK0305, and 18K08240).

## Conflict of Interest Statement

The authors declare that the research was conducted in the absence of any commercial or financial relationships that could be construed as a potential conflict of interest.
