# Supplementary material for: Therapeutic Silencing of Centromere Protein X Ameliorates Hyperglycemia in Zebrafish and Mouse Models of Type 2 Diabetes Mellitus
Source: Front Genet. 2019 Jul 29;10:693. doi: 10.3389/fgene.2019.00693 (PMC6681619; doi:10.3389/fgene.2019.00693)
Supplement: Supplementary file 1 [file Table_1.docx]

Supplementary Material

# Supplementary Data

**Real-time Quantitative Polymerase Chain Reaction (qPCR) Analysis**

Total RNA from tissues was extracted using Isogen (Nippon Gene, Tokyo, Japan) combined with the clean-up protocol of the RNeasy Mini kit (QIAGEN, Hilden, Germany). cDNA synthesis from 500 ng total RNA was performed using a ReverTra Ace qPCR RT Kit (Toyobo, Osaka, Japan). Real-time qPCR was performed with cDNA samples using Power SYBR Green Master Mix (Applied Biosystems, Foster City, CA, USA) and the ABI StepOnePlus Real-Time PCR System (Applied Biosystems) according to the manufacturer’s instructions. The relative mRNA levels were determined using 18S as an endogenous control gene.

**Morpholino Treatment**

Intraperitoneal injection of Morpholino (MO) was performed as previously described ([Kinkel et al., 2010](#_ENREF_1)). In brief, adult male fishes (AB strain) were anesthetized by temperature decrement using ice water (gradually from 17°C to 12°C). Body weight was measured, and the volume of MO solution was calculated to achieve a dose of 15 mg/kg fish weight, adjusted to 10 µL in distilled water. Thereafter, MO was intraperitoneally injected at a dose of 15 mg/kg fish weight. Fishes were recovered in fresh system water after intraperitoneal injection. MO treatment was performed twice a week during the 8-week experiment period.

**Preparation of *cenpx* sgRNA**

CHOP-CHOP web tool (http://chopchop.cbu.uib.no/) was used to design a single guide RNA (sgRNA) target site in the exon 3 of *cenpx* (Supplementary Table S1). The sgRNA for *cenpx* was synthesized by annealing a T7 promoter-containing gene-specific primer with sgRNA scaffold primers ([Yin et al., 2015](#_ENREF_2)). The annealed oligos were filled in with Taq DNA polymerase in the presence of 50 mM dNTP (New England Biolabs, Ipswich, MA, USA). The resultant double-stranded DNA (dsDNA) was used as a template for T7 RNA polymerase reaction using MAXIScript T7 kit (Invitrogen, Carlsbad, CA, USA). Purification of sgRNA was performed using RNA Clean & Concentrator columns (Zymo Research, Irvine, CA, USA). The RNA concentration was determined, and the RNA quality was evaluated using agarose gel electrophoresis.

**Preparation of Genomic DNA, Heteroduplex Mobility Assay (HMA) and Sequencing Analysis**

To prepare genomic DNA, the un-injected and *cenpx* sgRNA/Cas9-injected embryos were grown to 24 hpf (hour post fertilization), and 8-10 embryos were picked up for DNA isolation. The genomic DNA was extracted using 20 μL/embryo of 50 mM sodium hydroxide (NaOH) for 20 min at 95°C. The sample was mixed with 4 μL of 1 M Tris-HCl to adjust the pH. Genomic fragments at the target sites were amplified by PCR with GoTaq® Hot Start Polymerase (Promega, Fitchburg, WI, USA) using the locus-specific primers (Supplementary Table S1). The PCR amplicons were electrophoresed on a 10% polyacrylamide gel (Wako Pure Chemicals, Osaka, Japan). To identify the genome modification induced by *cenpx* sgRNA/Cas9, F0 founders were outcrossed to wild type fish and the genomic DNA was extracted from tailfin clips of adult F1 fish using the protocol described above. DNA from individual fish was used as a template for subsequent PCR (primer sequences are described in Supplementary Table S1) and the sequences of PCR amplicons were analyzed by the Center of Genetics (Mie University, Japan).

***In vitro* Silencing of *Cenpx* Gene**

Mouse Hepa 1-6 hepatoma cells (RIKEN Cell Bank, Tsukuba, Ibaragi, Japan) were cultured in Dulbecco’s modified Eagle’s medium (Life Technologies, Carlsbad, CA, USA) supplemented with 100 μg/mL of streptomycin sulfate (Sigma–Aldrich, St. Louis, MO, USA), 100 U/mL penicillin G (Sigma–Aldrich) and 10% fetal bovine serum (Life Technologies, Carlsbad, CA, USA) and maintained at 37°C with 5% CO2 and 95% air. The cells were transfected at 70% confluency with 75 pmol of each siRNA using Lipofectamine 3000 reagent (Life Technologies) in a six-well plate format. Two days after siRNA transfection, the total RNA was purified using RNeasy Mini Kit (Life Technologies). First-strand cDNA was prepared with 200 ng of total RNA using ReverTra Ace qPCR RT Master Mix (Toyobo, Osaka, Japan). qPCR was performed with Power SYBR Green Master Mix (Life Technologies) in triplicates, according to the manufacturer’s protocols.

**Reference:**

Kinkel, M.D., Eames, S.C., Philipson, L.H., and Prince, V.E. (2010). Intraperitoneal injection into adult zebrafish. J Vis Exp.

Yin, L., Maddison, L.A., Li, M., Kara, N., Lafave, M.C., Varshney, G.K., et al. (2015). Multiplex Conditional Mutagenesis Using Transgenic Expression of Cas9 and sgRNAs. Genetics 200, 431-441.

# Supplementary Figures

**Supplementary Figure S1.** Effects of *cenpx* MO and *hmox1* MO on body weight and fasting blood glucose in DIO AB strain zebrafish. (A) Changes in body weight in each group during the 8-week overfeeding experiment. (B) Fasting blood glucose level in each group. n = 10; mean ± SE. **P* < 0.05, ***P* < 0.01 versus DIO group.


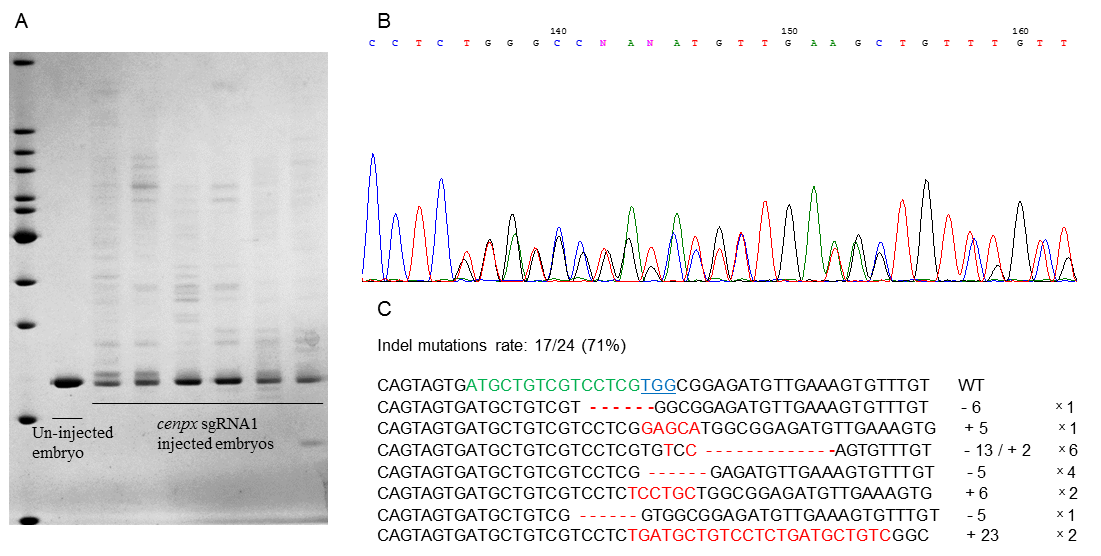


**Supplementary Figure S2.** Identification of the mutations induced by *cenpx* sgRNA/Cas9. (A) Heteroduplex mobility assay (HMA) showing efficient *cenpx* mutagenesis in cas9/sgRNA transgenic larvae at 1 dpf (day post fertilization). (B) Chromatograms showing sequence analysis to confirm heterozygous mutation in the targeted region of *cenpx*. (C) Sequences of *cenpx* mutations in 24 F1 individuals. The wild-type sequence is shown at the top, with the targeted genomic sequences and PAM sequences shown by the green and blue letters, respectively. The deleted and inserted nucleotides compared with the wild-type sequence are indicated by the red dashes and red letters, respectively. The numbers of nucleotides deleted (-) and inserted (+) are indicated to the right with the detection number.

**Supplementary Figure S3.** Knockdown efficacy of *Cenpx* siRNAs was measured by qPCR using Hepa 1-6 cells. n = 3; mean ± SE. **P* < 0.05, ***P* < 0.01 versus the control group.


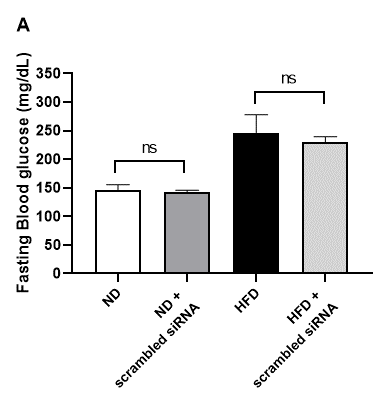

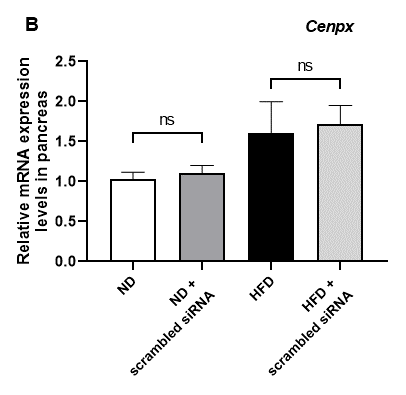


Supplementary Figure S4. Effects of scrambled siRNA on fasting blood glucose and *Cenpx* mRNA levels in mouse. (A) Changes in in fasting blood glucose levels. (B) Relative mRNA levels in the pancreas tissues of mice injected with scrambled siRNA. n = 5; mean ± SE. ns, not significant.


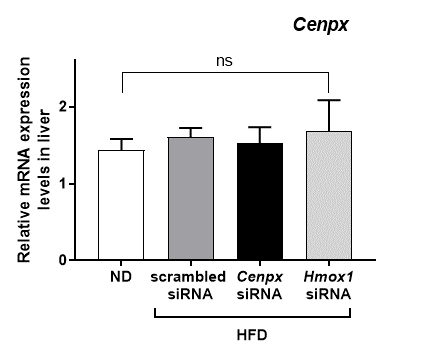


**Supplementary Figure S5.** Relative mRNA levels in the liver tissues of mice injected with *Cenpx*-siRNA/Hmox1-siRNA. ND, normal diet; HFD, high-fat diet. n = 5. ns, not significant versus the ND group.


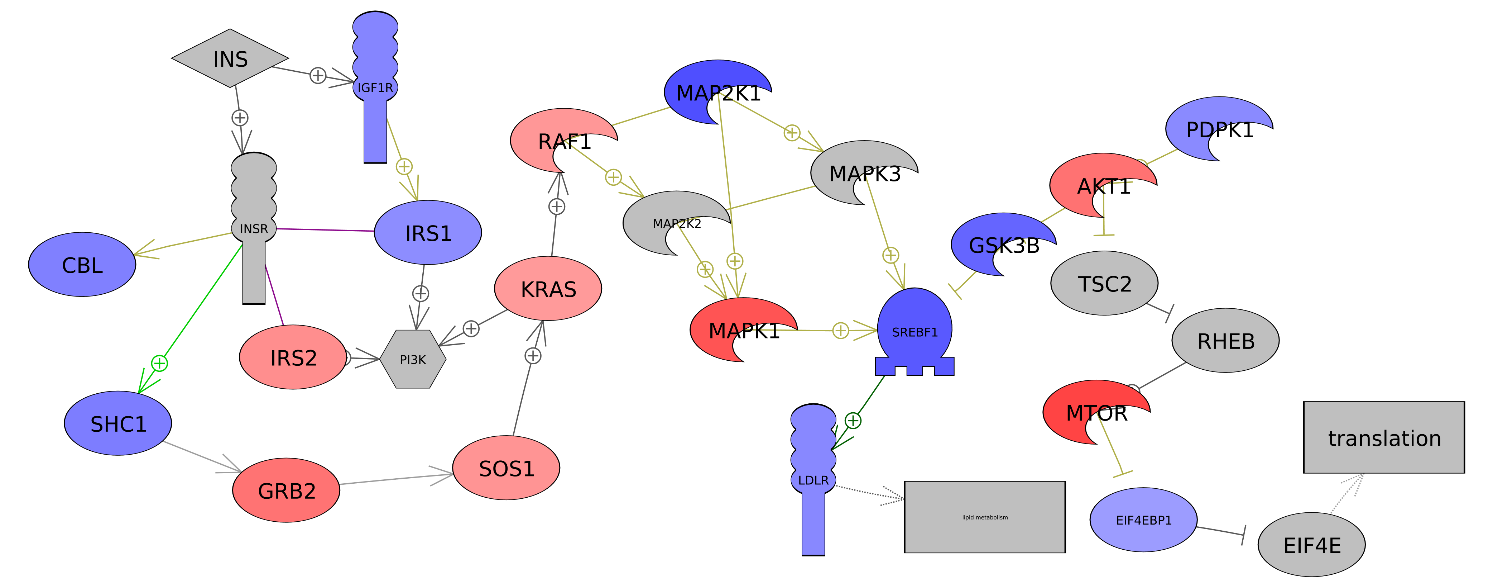


**A**

**B**


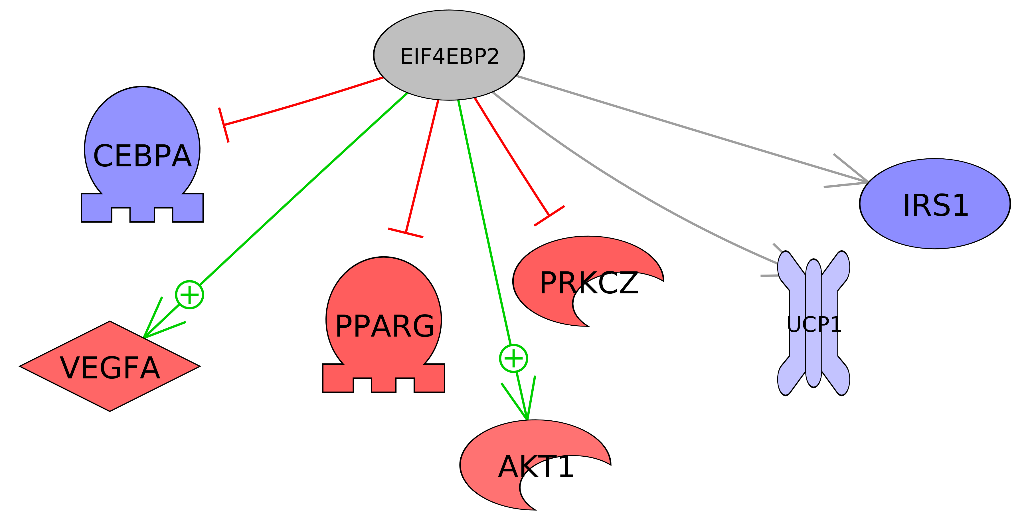


**Supplementary Figure S6.** (A) Upregulated INSULIN signaling pathway by *Cenpx* knockdown in T2DM mice as compared with control T2DM mice. (B) Upregulated EIF4EBP2 pathway acting downstream of mTORC1 after *Cenpx* knockdown in T2DM mice as compared with control T2DM mice. Red and blue denote genes with increased and decreased expression, respectively, in *Cenpx* knockdown HFD mice as compared with control HFD mice. Grey indicates the genes that were undetected in the gene expression analysis.
